# Global and Local Deformation Effects of Dry Vacuum-Consolidated Triaxial Compression Tests on Sand Specimens: Making a Database Available for the Calibration and Development of Forward Models

**DOI:** 10.3390/ma15041528

**Published:** 2022-02-18

**Authors:** Zenon Medina-Cetina, Ahran Song, Yichuan Zhu, Alma Rosa Pineda-Contreras, Amy Rechenmacher

**Affiliations:** 1 Zachry Department of Civil and Environmental Engineering, Texas A&M University, College Station, TX 77843-3136, USA; 2 Mott MacDonald Japan, 4F Nihonbashi Honcho 1-Chome Building, 1-9-13 Nihonbashi Honcho, Chuo-ku, Tokyo 103-0023, Japan; ahran.song@mottmac.com; 3 Civil & Environmental Engineering Department, Temple University, Philadelphia, PA 19122-6018, USA; yichuan.zhu@temple.edu; 4 Laboratorio de Geoinformática, Instituto de Ingeniería, Universidad Nacional Autónoma de México UNAM, Torre de Ingeniería 2° Piso, Circuito Escolar C.U., Coyoacan, Mexico City 04510, Mexico; apinedac@iingen.unam.mx; 5 Sonny Astani Department of Civil & Environmental Engineering, University of Southern California, Los Angeles, CA 90089-2531, USA; arechenm@usc.edu

**Keywords:** experimental database, sand triaxial compression tests, 3D-DIC, localization effects, forward models, numerical models, specimen heterogeneity

## Abstract

A comprehensive experimental database containing results of a series of dry vacuum-consolidated triaxial compression tests was populated. The tests were performed on sand specimens and conducted under similar experimental conditions, in which specimens’ boundary deformation was captured using a three-dimensional digital image correlation analysis (3D-DIC). The use of a standard triaxial device along with the 3D-DIC technology allowed the specimens’ global and local boundary displacement fields to be computed from start to end of the compression phase. By repeating each test under the same experimental conditions and building the specimens using the same type of sand, the boundary deformation patterns could be identified, and the statistics associated with both global and local displacement fields could be assessed. Making this experimental database available to others should serve to calibrate as well as develop new forward models to account for effects associated with the specimens’ local displacements and material heterogeneity and include statistics to represent a specimen’s random response. Moreover, this work will serve as a basis for the statistical characterization of spatio-temporal boundary localization effects used to develop stochastic models and machine-learning models, and simulate virtual triaxial tests.

## 1. Introduction

An experimental database was populated from a series of dry vacuum-consolidated triaxial compression tests performed on sand specimens tested under similar experimental conditions; boundary deformation was captured by the use of a three-dimensional digital image correlation analysis (3D-DIC). Capturing high-resolution displacement fields on a sample’s boundary makes possible identification and characterization of local deformation effects, such as strain localization, which we hypothesize controls the failure mechanisms of geotechnical structures across multiple scales, from microcracking of material to structural failures of foundations, dams, excavations, and anchoring systems. This study of localization effects in sands under laboratory-controlled conditions thus provides significant evidence of specimens’ multiscale behavior, which plays a key role in explaining the evolution of micro- to macro-deformation patterns under different loading and boundary conditions, and for a given set of material physical properties, such as particle size, particle angularity, density, stiffness, and strength [1,2].

The mechanical response of granular materials, including the effect of material density and granular packing within a specimen, can be investigated using gamma rays [3,4], X-ray computed tomography [5,6], and micro-focused X-ray systems [7,8,9]. Advances in the use of these full-body scanning techniques have allowed the morphology of each particle to be characterized by a particle’s trajectory under laboratory compression conditions. From a micro-scale standpoint, these techniques have allowed granular properties of the material to be characterized and the development of the material failure mechanisms to be accurately captured.

Digital image correlation has emerged as an optical non-intrusive experimental technique to carry out full-field deformation measurements by capturing high-resolution displacement fields over the boundary of a specimen during the loading phase. This methodology requires a computational algorithm to assimilate consecutive digital images used to estimate the corresponding displacement fields [10]. DIC has been used in geoengineering as an analysis tool with some application in the analysis of arching phenomena in soil [11] and the load distribution in foundation beds [12]. When applied to sand specimens under plane strain conditions, DIC defined 2-D kinematic behavior in terms of shear and compression bands, characterized by thickness and inclination [13,14]. DIC also identified the evolution of “force chains” and “vortex-type” structures through the course of the specimen’s deformation [15,16]. The same methodology but in 3-D was applied by Rechenmacher and Medina-Cetina [17,18] to a limited set of dry vacuum-consolidated specimens; at the time, Rechenmacher and Medina-Cetina [17,18] focused on the influence of boundary conditions and specimen heterogeneity in the definition of only elastic constitutive parameters by the solution of the inverse problem.

Most previous DIC studies performed to characterize geomaterials focused on kinematic effects associated with various experimental conditions [19,20,21,22], including grain morphology, stiffness, strength, sample preparation, and effect of boundary conditions [18,23]. The combined effects of these control parameters led to varying experimental responses even under the same experimental conditions, which motivated populating a comprehensive database that would explore the combined impact of all aforementioned properties, making available both global and local deformation effects, as well as its uncertainty. The experimental database introduced in this paper challenges traditional calibration of forward models that make use of only global triaxial measurements provided by the standard triaxial device, in which the specimen is assumed to be homogeneous, to have perfect cylindrical geometry, and that all boundary effects acting on the sample are already accounted for.

Control variables included in the experimental design of this database are sample density, confining pressure, sample preparation method, initial geometry, and repeatability and reproducibility. Repeatability was a unique consideration to allow for the identification and characterization of global and local deformation patterns, and to assess the corresponding uncertainty of both global and local effects while conducting the same experiment under the same experimental conditions (i.e., same operator, same equipment, same material, same testing procedures). Capturing both global and local deformation effects also allowed for accounting for multiscale effects associated with micro- and meso-scale behavior, which can be used when developing a multiscale forward model [19,20,21].

All data used in this paper, including readings from the triaxial device (global re-sponse) and boundary displacement fields (local response) as captured by the 3D-DIC, are available at Supplementary Material.

## 2. Soil Experiments

**Figure 1:**
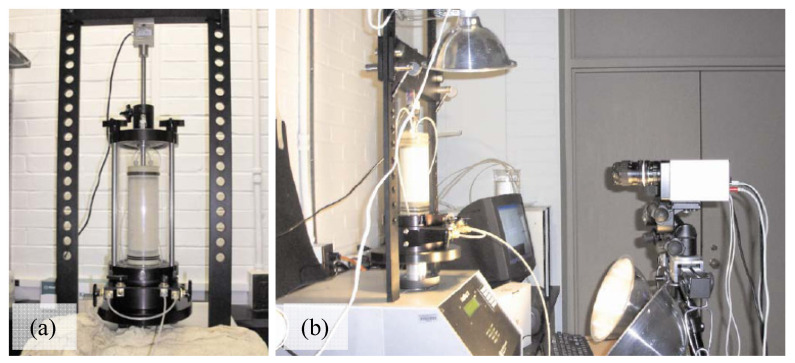
Triaxial system (**a**), and 3-D imaging system (**b**).

**Figure 2:**
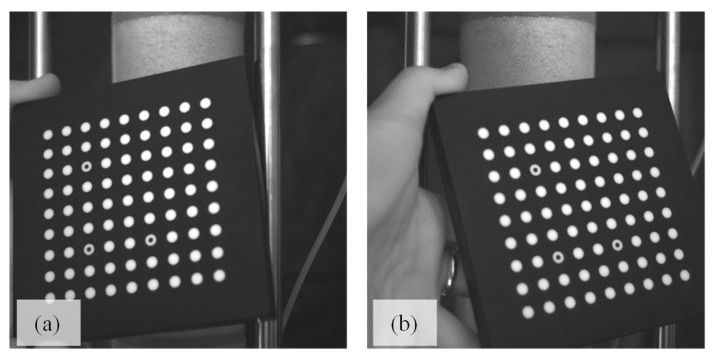
Camera calibration grid as captured in the left (**a**) and right image (**b**).

The testing technique used to develop the database is similar to that used in conventional triaxial compression tests, except the triaxial plexiglass cell was removed to avoid light reflection and refraction, which would likely cause distortion during the capture of stereo-digital images. For this reason, specimens were vacuum-consolidated instead of by a fluid-induced consolidation. An automated triaxial device was used for the compression tests and to measure the global stress-strain axial response. The triaxial frame and triaxial 3-D imaging systems setup are presented in Figure 1. The 3-D imaging system used to capture the boundary displacement fields consists of two digital cameras positioned 25 cm from each other and 50 cm away from the sample. The digital cameras, Q-IMAGING PMI-4201 (Correlated Solutions: Irmo, SC, USA), were equipped with experimental-grade, charge-coupled device sensors that were calibrated through analyzing synchronous images of a standard grid oriented at different angles, as shown in Figure 2. Before each specimen was loaded, the camera was calibrated to define the 3-D spatial framework and the required parameters to compute the displacement fields during the specimen’s compression [24].

The material selected for testing was a dry sand, classified as SP with Cu = 2.34 and Cc = 1.11, which provided a distinct grain-color spectrum suitable for pattern recognition during imaging analysis (as seen behind the sample’s latex membrane). The sand specimen was constructed using a standard cylindrical mold following two preparation methods: vibratory compaction and dry pluviation. The experimental design produced a series of heterogeneous specimens (compacted layers through vibratory compaction) and a series of homogeneous specimens (one single layer through dry pluviation). Each specimen setup was made on the triaxial frame, the mold was then removed, and an isotropic vacuum pressure was applied to the base of the specimen to maintain its effective stress integrity, by the use of a vacuum pump at a prescribed pressure. The specimen then was loaded from the bottom at a controlled deformation rate of 0.2% of axial strain/min, and stereo-digital images were taken at every 0.05% of axial strain.

All tests’ coding and measures of the physical characteristics defining the control variables included in the database are presented in Table 1 and Table 2, corresponding to specimens prepared following vibratory compaction (multiple-layered heterogeneous specimens) and dry pluviation methods (single-layer homogeneous specimens), respectively. A total of 27 specimens were tested, comprising 25 specimens of nominally similar density (heterogeneous and homogeneous combined), one two-layer half-loose and half-dense specimen, and one loose sand specimen. Most specimens were consolidated at 40 kPa of vacuum pressure, but one specimen was consolidated at a confining pressure of 20 kPa and two were consolidated at 60 kPa. The average diameter of all specimens is 71.15 mm, with a standard deviation of 0.27 mm; the average height is 158.31 mm, with a standard deviation of 1.62 mm. The dense specimens’ average initial density is 1712.89 kg/m^3^, with a standard deviation of 10.10 kg/m^3^; the average relative density is 91.72%, with a standard deviation of 3.43%. Eighteen samples were prepared in three layers by the vibratory compaction method (heterogeneous) and eight samples were prepared by the dry pluviation method (homogeneous). One specimen was built in two compacting layers; the lower layer was dense, with a relative density of 98.87%, and the upper layer was loose, with a relative density of 30.54%.

**Figure 3:**
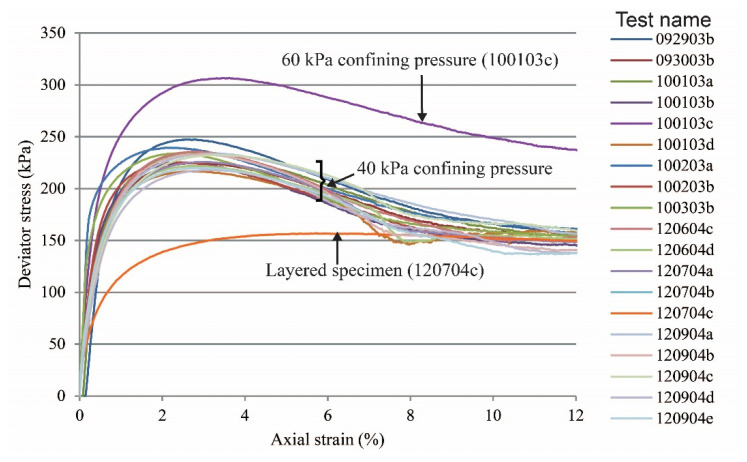
Global stress-strain responses of sand specimens prepared by the vibratory compaction method.

**Figure 4:**
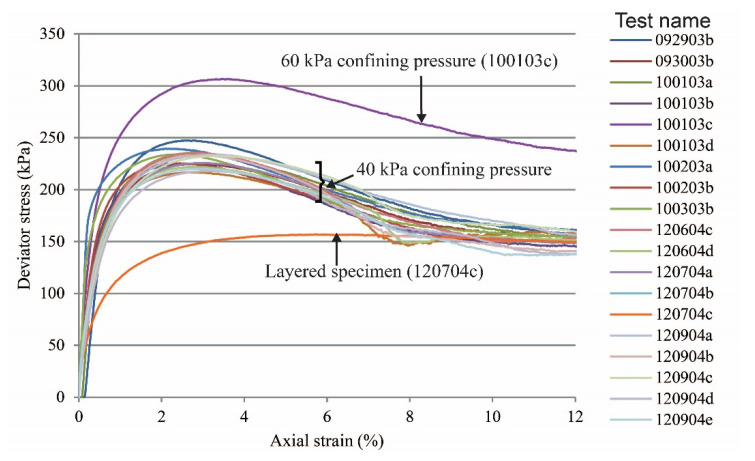
Global stress-strain relations of sand specimens prepared by the dry pluviation method.

Global stress-strain curves for all tests are presented in Figure 3 (vibratory compaction) and Figure 4 (dry pluviation). Both figures show that for dense sand specimens with a confining pressure of 40 kPa, the maximum deviatoric stress oscillated between 220 and 255 kPa and the deviatoric stress at the critical state ranged between 140 and 160 kPa. For the two tests with a confining pressure of 60 kPa, the deviatoric stresses were 306 and 345 kPa at peak and 231 and 225 kPa at critical state. We hypothesize that deviations in deviatoric stress with the same confinement condition were caused by the variation of relative density within the specimen as a result of the sample preparation methods. The loose specimen test had no peak stress and yields at 142 kPa and 3.2% of axial strain and reached critical state at about 148 kPa. The two-layered specimen test did not have a behavior typical of a dense sand specimen, but instead had a response similar to that of a loose sand specimen, yielding at 162 kPa at 4.0% of axial strain and reaching critical state at about 168 kPa.

**Figure 5:**
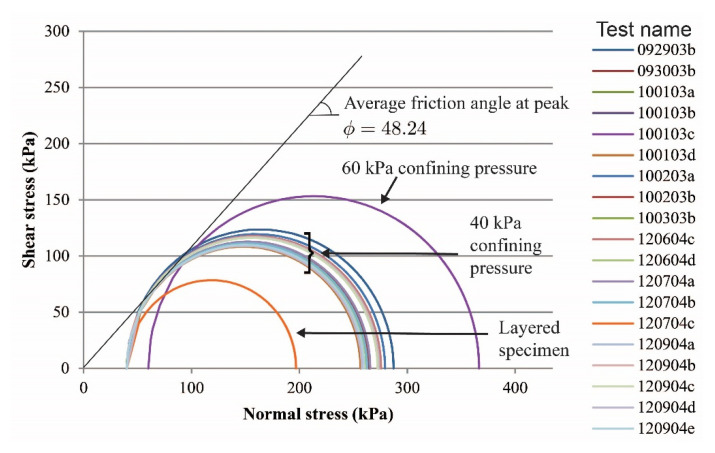
Mohr’s circles of sand specimens prepared by the vibratory compaction method.

**Figure 6:**
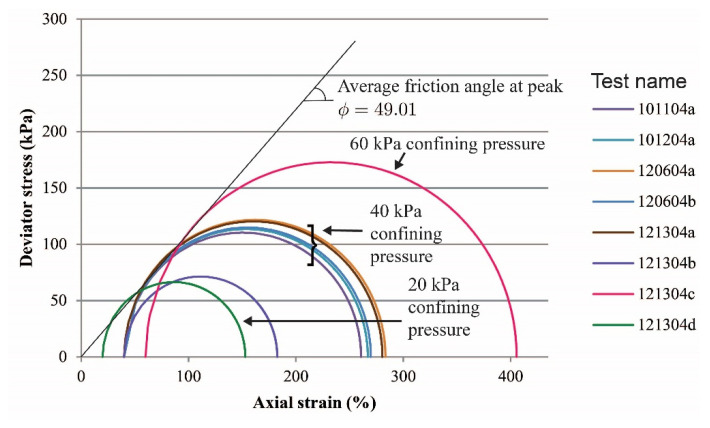
Mohr’s circles of sand specimens prepared by the dry pluviation method.

**Figure 7:**
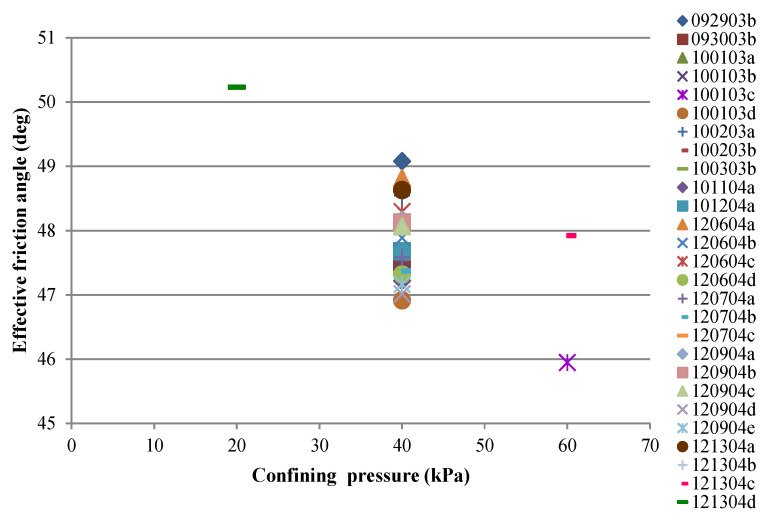
Effective friction angles at failure for dense specimens.

Mohr’s circles for experiments on all sand specimens are presented in Figure 5 and Figure 6. Failure envelopes and friction angles at the peak strength for dense sand specimens are also shown. A summary of the point of estimates of friction angles versus confining stress for all dense specimens regardless of sample preparation is presented in Figure 7, where the mean and standard deviation of the friction angle for all dense specimens confined at 40 kPa are 47.86 and 1.81, respectively.

## 3. 3-D Digital Image Correlation Analysis (3D-DIC)

**Figure 8:**
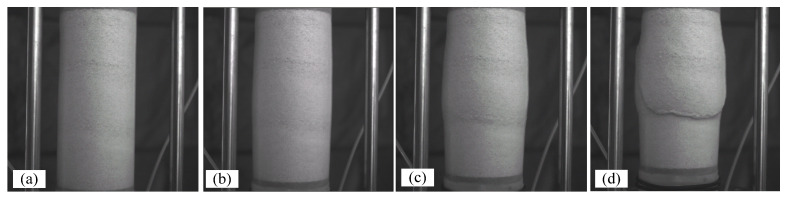
Photographic images of test 120904c at 0.2% of axial strain (**a**), 3.6% of axial strain (**b**), 7% of axial strain (**c**), and 12% of axial strain (**d**).

Digital image correlation is a non-destructive optical approach that permits the estimation of high-resolution (micro) displacement fields over the surface of a specimen during loading and consequently capturing local deformations effects, such as shear and compression bands. Three-dimensional digital image correlation, or 3D-DIC, was developed based on principles analogous to the human eye’s depth perception: stereo-digital images are taken during loading at a constant frequency in order to reconstruct the displacement field over a segment of the surface of the cylindrical specimen by tracing pixel patterns along sequences of images. During the course of shearing, two 14-bit Q-IMAGING PMI-4201 (Correlated Solutions: Irmo, SC, USA), digital cameras with 4.2 megapixels of resolution (2024 × 2024 pixels) took simultaneous images every 15 s, corresponding to 0.05% of axial strain during the triaxial compression test. Figure 8 illustrates one sequence of deformation; images that are taken by one digital camera capture the state of the specimen at the deformation stages of 0.2%, 3.6%, 7%, and 12% of axial strain, presenting the state of the sample at the elastic zone, peak stress, softening zone, and critical state, respectively.

**Figure 9:**
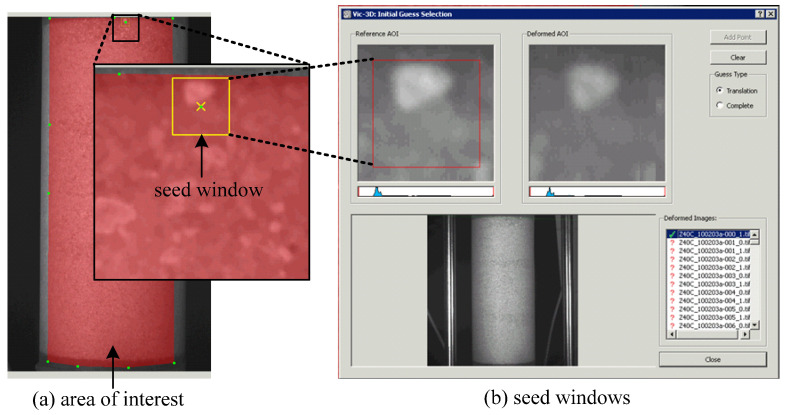
Reference image: area of interest (**a**) and seed windows as produced by (VIC-3D) (**b**).

VIC-3D software was used to digitize the photo images and assimilate the images into 3-D full-field displacements [25]. The first step is to select an area of interest on an image of reference and define a seed window at the first set of images (undeformed configuration). An area of interest is defined for which the displacements will be computed, and a seed window is set at the common pixels that are clearly identified in both left and right images, as shown in Figure 9. A subset of 45 pixels and a step size of 3 pixels were selected to achieve as close to grain-scale resolution in the displacement measurement as possible.

**Figure 10:**
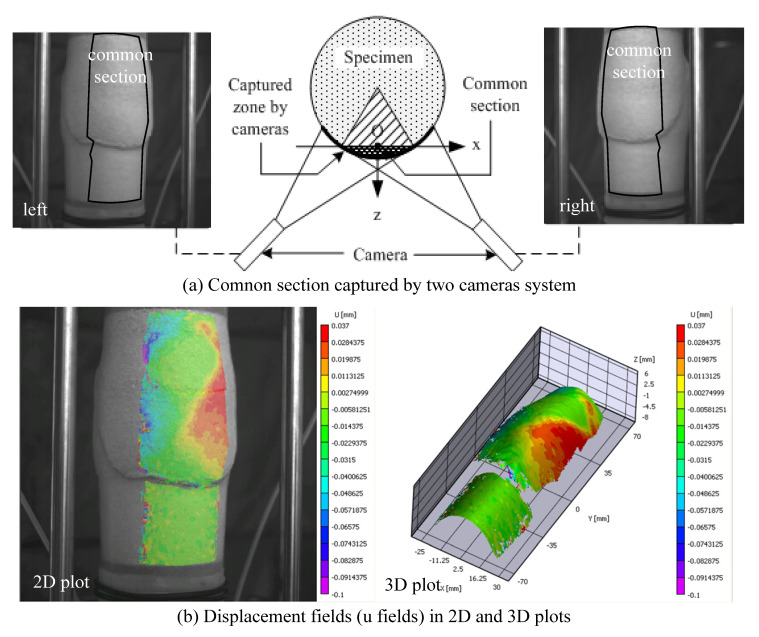
Common section captured by the VIC-3D two-camera system (**a**) and displacement fields in 2-D and 3-D (**b**).

Once the 3-D imaging system is calibrated and the reference image is prepared for analysis, VIC-3D can perform the 3-D surface reconstruction. The common section captured by two cameras is where the displacement fields are analyzed, which accounts for up to one-fourth of the circumference of the specimen (Figure 10a). VIC-3D generates 2-D and 3-D contour plots of the displacement fields available in the common section, where the displacement fields are computed. Some limitations on the use of this technology include cases when it is not possible to follow pixels from one set of stereo-images to the next, which may happen due to light reflection, or at late stages of deformation where anomalies form on the fully sheared surface of the specimen, limiting the view from either of the cameras. For instance, Figure 10b shows a 2-D and 3-D horizontal displacement field reconstruction at 12% of axial strain for test 120904c, where some segments of data are lost, indicating that the use of the data will be limited.

**Figure 11:**
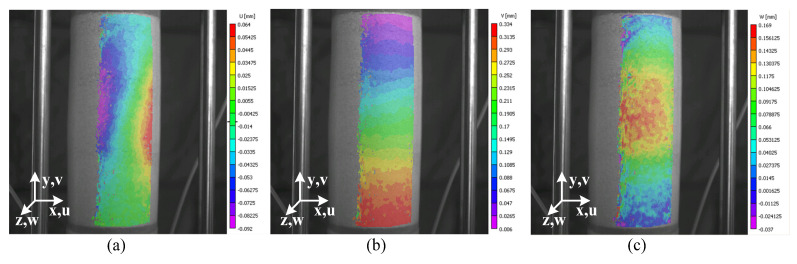
State of deformation of test 120904c between 2.8% and 3.0% of axial strain, obtained by the 3D-DIC process: *u* field = horizontal (**a**), *v* field = vertical (**b**), and *w* field = out-of-plane (**c**).

Figure 11 illustrates a state of deformation showing displacement fields at 3.0% of axial strain when the reference image is the image at 2.8% of axial strain. The orientation of the system setup is described in a global Cartesian system, and *u*, *v*, and *w* are displacement components corresponding to the *x*, *y*, and *z* directions, respectively.

**Figure 12:**
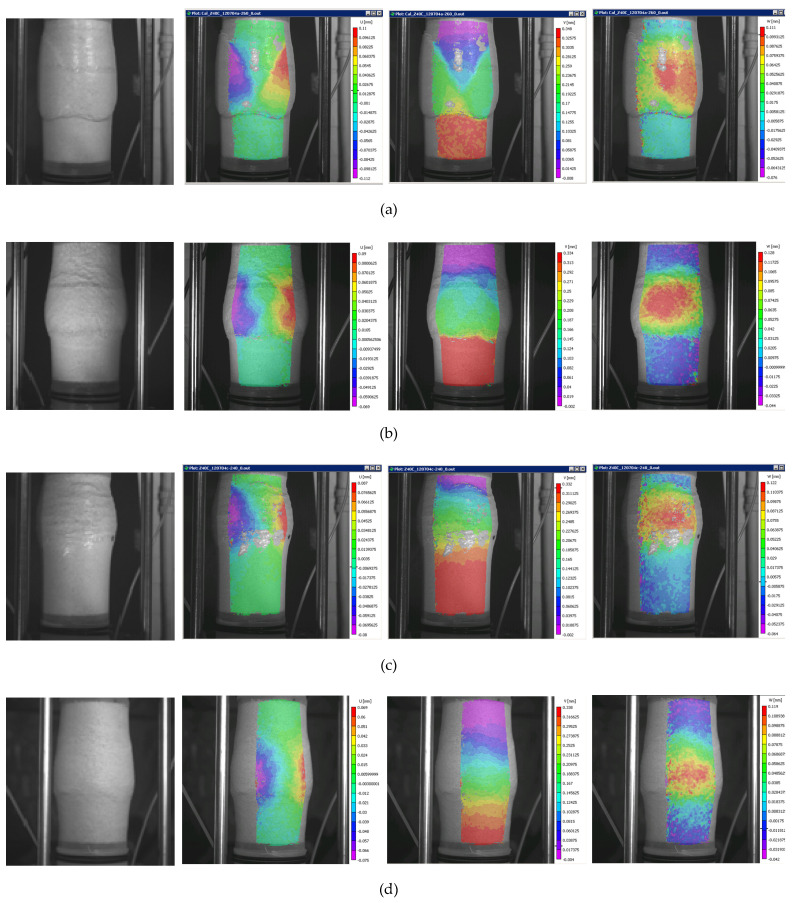
Different localization effects at failure. Digital images and corresponding displacement fields *(u*, *v*, and *w)* at 12% of axial strain from tests 120704a (**a**), 100103c (**b**), 120704c (**c**), and 121304b (**d**).

As reported in Alshibli et al. [5] and Desrues and Viggiani [26], strain localization in sand can be manifested in a wide range of phenomena, from an undeformed configuration to a deformed configuration, in which compression, expansion, and rotation over the sample specimen can be observed, as was identified in the present study. Figure 12 presents four distinct failure modes as noted in tests 120704a, 100103c, 120704c, and 121304b at 12% of axial strain. The deformation patterns show a variety of boundary phenomena, from V-shaped shear-band formations to bulging in different places. Figure 10, Figure 11 and Figure 12, are screen captures that allow to see both the specimen and the displacement field components overlaying it, illustrating the extent of the digital image analysis, the different deformation patterns, and the challenge of data curation for some of them because of lighting reflection. Notice that some of the associated colorbars showing the order of magnitude of the displacement field components may not be fully readable since these figures were screen captures. However, reproducible data corresponding to all tests presented in this paper are publicly available and accessible at https://dataverse.tdl.org/dataverse/SGL-MDPI-Topic-StochasticGeomechancis-ForwardModeling (last accessed on 1 December 2021).

**Figure 13:**
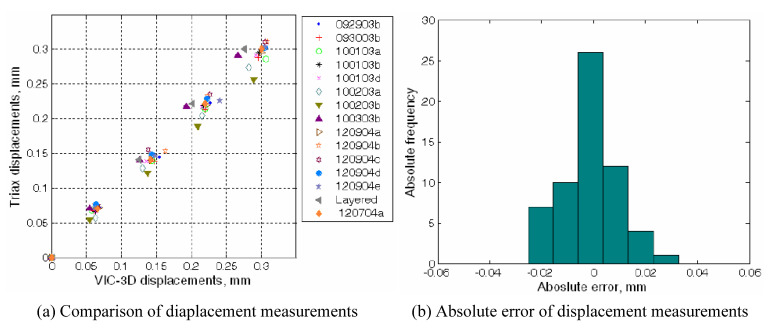
Accuracy analysis: Comparison of displacement measurements between the triaxial device LVDTs and VIC-3D displacement readings (**a**) and frequency histogram of absolute error of displacement measurements between the triaxial test system and VIC-3D (**b**).

The accuracy of displacement measurements produced by DIC was validated by comparing vertical displacements between DIC and LVDT observations at the loading frame, as shown in Figure 13a, corresponding to the elastic deformation range. The absolute difference between the triaxial device’s and DIC’s measurements in the vertical direction had a mean absolute error of 0.00 mm and a standard deviation of 0.02 mm (metric of accuracy), which is consistent with the order of magnitude observed in biaxial tests using only one camera for both vertical and horizontal displacements [27]. Previous research [28] suggests that the accuracy of the horizontal and out-of-plane displacements should be of the same order as that of vertical displacements.

## 4. Post-Processing of Image Data

### 4.1. Piece-Wise Integration of Cumulative Displacement Fields

In 3D-DIC, displacement fields are computed by assessing the image differences of consecutive images. The strategy set to secure the computation of each displacement field obtained from VIC-3D is to conduct incremental displacement field analyes every fourth set of images (i.e., every 0.2% of axial strain). Therefore, the “memory” of the spatial framework is lost after every fourth image. This frequency was set to retrieve the most information from the sequence of images (the farther the image analyzed, the less correlation is found with respect to the reference image). To elicit cumulative displacement fields with respect to the first reference image (i.e., undeformed state at zero strain), consecutive displacement fields had to be integrated.

**Figure 14:**
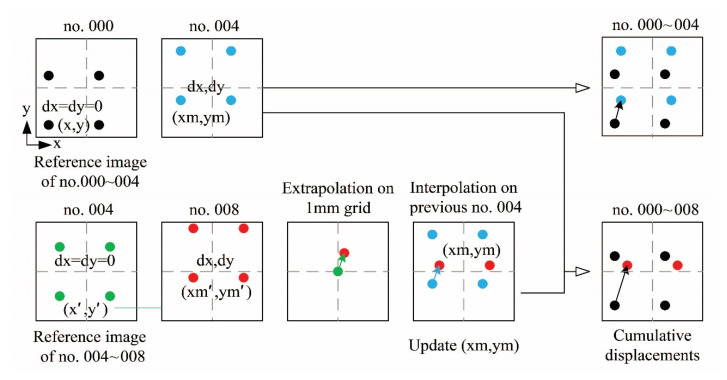
Scheme of interpolation and extrapolation for integration of cumulative displacement fields.

Figure 14 illustrates the integration flow-chart analysis as applied from image no. 000 to image no. 008, which corresponds to 0.4% of axial strain (i.e., two sequences of analysis). The reference image for no. 000–004 is image no. 000, and for the no. 004–008 is image no. 004. To accumulate displacements from image no. 000 to no. 008, the reference points of displacements between image no. 004 to no.008 were first interpolated on the last step’s spatial coordinates (image no. 004). After the interpolation, the sum of the current displacement (image no. 004 to 008) and the previous step’s displacements (image no. 000 to 004) becomes the cumulative displacement from image no. 000 to no. 008. This integration method was iteratively applied to all deformation sequences (sets of four images) from the undeformed stage to the last image set of all the compression tests.

### 4.2. Geometrical Transformation

**Figure 15:**
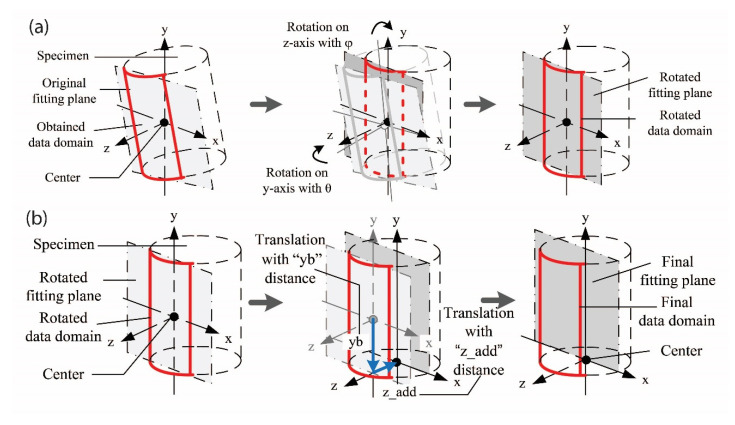
Schematic illustration of 3-D transformation: rotation (**a**) and translation (**b**).

Sutton et al. [28] and Lava et al. [29] reported that 3D-DIC system experiments had errors traced to sources, such as cross-camera matching, camera calibration, multiple correlation runs, and triangulation when integrating segments of the displacement fields. In this study, the images obtained from VIC-3D have already been corrected for data alignment as part of the calibration procedure. From the calibration images, various system parameters, including camera-based parameters, such as focal length, image center, lens distortion, and the relative orientation of the two cameras in space are computed. The resulting plotted shape of the 3-D image data on the Cartesian coordinate system was still not fully aligned with the orthogonal axes *(x*, *y*, and *z*), however. To correct this problem, a 3-D geometric rotation and translation were conducted, aimed at finding the best starting material coordinates for each test. The transformation procedure consisted of: (1) computing the coefficients of a best-fit plane by regression of all data points, (2) rotating the initial data points with the angles calculated from the relationship between a normal vector of a best-fit plane and the *y-* and *z-*axes (Figure 15a), (3) after rotations, translating the data points along *y-* and *z-*directions in order to align the data points consistent with the orthogonal coordinate system located at the center and base of the specimen (Figure 15b).

## 5. Results: Displacement Fields

### 5.1. 3-D Displacement Field under Cartesian and Cylindrical Coordinates

**Figure 16:**
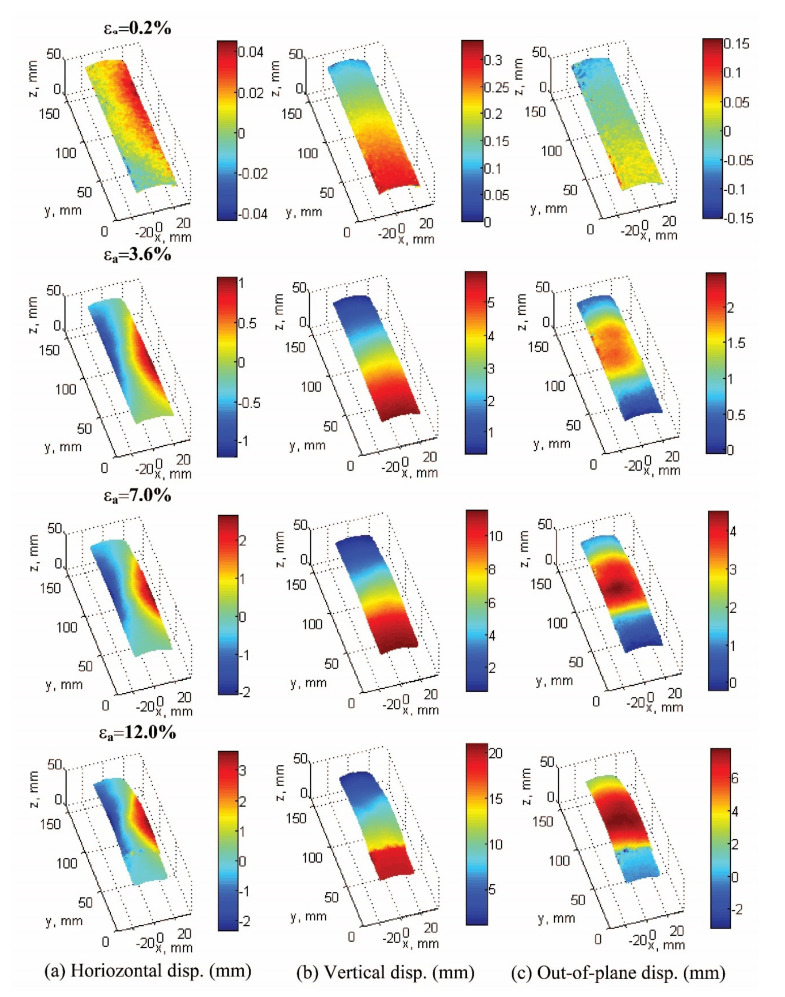
Cumulative displacement fields of test 120904c using Cartesian coordinates at 0.2%, 3.6%, 7%, and 12% of axial strain: horizontal displacement field *u* (**a**), vertical displacement field *v* (**b**), and out-of-plane displacement field *w* (**c**).

**Figure 17:**
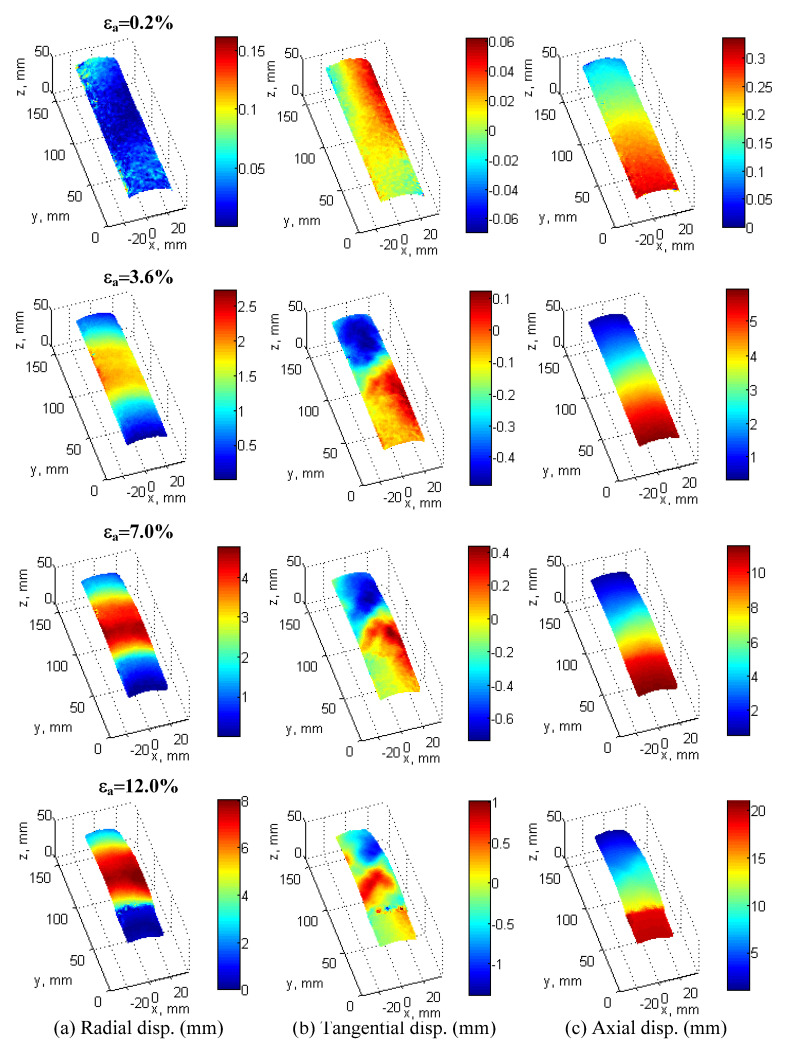
Cumulative displacement fields of test 120904c using cylindrical coordinates at 0.2%, 3.6%, 7%, and 12% of axial strain: radial displacement field (**a**), tangential displacement field (**b**), and axial displacement field (**c**).

Figure 16 and Figure 17 are introduced to illustrate typical displacement fields under Cartesian and cylindrical coordinates. These were captured at 0.2% (elastic zone), 3.6% (peak stress), 7% (softening zone), and 12% (critical state) of axial strain, respectively. A graphical explanation of how the cylindrical displacements are computed can be found in [30].

Under Cartesian coordinates (Figure 16), horizontal displacement fields show a developing persistent shear band clearly starting from 3.6% of axial strain. Vertical displacement fields at the bottom of the specimen correspond to global displacement (e.g., compression loading is induced from the bottom of the specimen), which follows the displacement loading rate with 0.2% of axial strain/min. The out-of-plane displacement fields show a bulging zone that becomes evident after 3.6% of axial strain.

Figure 17 presents the same information as in Figure 16, but in cylindrical coordinates, where radial displacement fields better represent the bulging effect of the specimen. Figure 16c shows that after peak stress (3.6% of axial strain), there is a clear pattern of displacement fields between the bottom segment and the rest of the specimen, with a rigid block type of movement at the bottom, which can be associated with higher local density and the effect of the boundary conditions.

**Figure 18:**
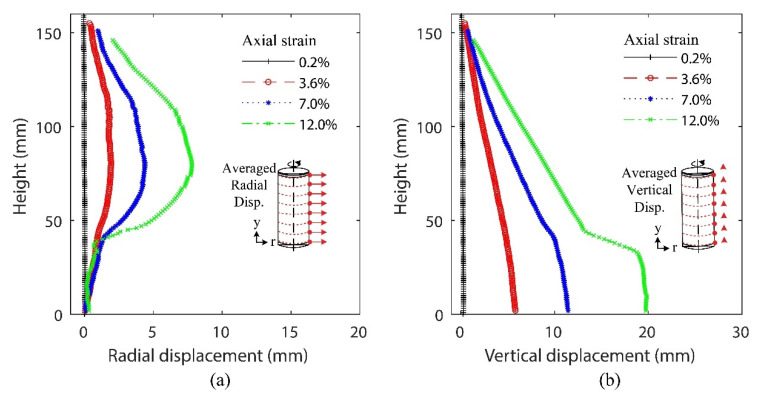
Vertical profiles of averaged displacements along the axial direction of test 120904c: radial displacements (**a**) and vertical displacements (**b**).

A simplified representation of the displacement fields generated by 3D-DIC for the same test presented above is shown in Figure 18, which will be used to present a first description of the boundary data. This simple representation is suitable for the calibration of orthotropic numerical models, which is hypothesized to be the least computationally expensive approach to start exploring calibrations of forward models for sands. The proposed approach presents two vertical profiles of the averaged radial (a) and vertical (b) displacements computed across the same height of the sample’s surface (Figure 18). Both vertical profiles show displacements that correspond to homogeneous deformations between 0.2% and 3.6% of axial strain, but not after 7% of axial strain. As the shearing progresses, a nonsymmetric response is expected after the peak stress, which can be observed in both the radial and vertical displacement fields. For this particular test, both vertical profiles show that the bottom segment of the specimen follows a distinct pattern in which it is separated from the bulging surface of the specimen by shearing, causing it to move vertically as a single rigid block.

### 5.2. 1-D Radial and Vertical Displacement Fields (Vertical Profiles)

**Figure 19:**
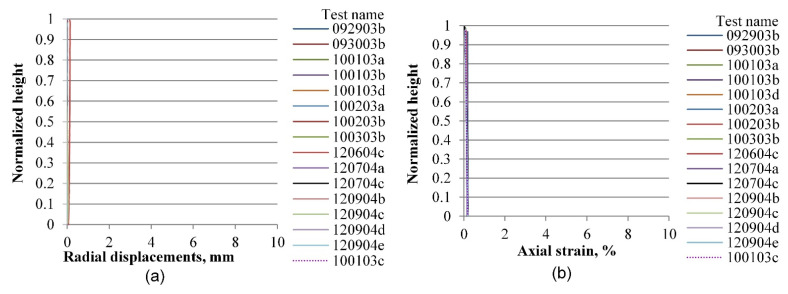
Vertical profiles of averaged displacements at 0.2% of the axial strain of sand specimens prepared by the vibratory compaction method: radial displacements (**a**) and vertical displacements (**b**).

**Figure 20:**
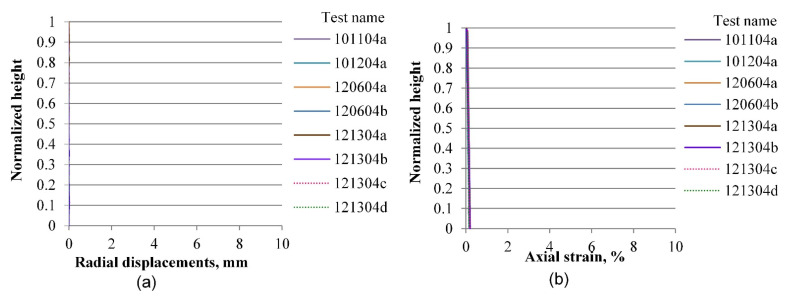
Vertical profiles of averaged displacements at 0.2% of the axial strain of sand specimens prepared by the dry pluviation method: radial displacements field (**a**) and vertical displacements field (**b**).

**Figure 21:**
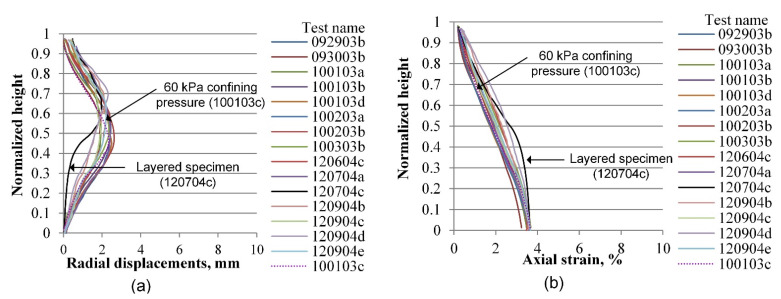
Vertical profile of averaged displacements at 3.6% of the axial strain of sand specimens prepared by the vibratory compaction method: radial displacements field (**a**) and vertical displacements field (**b**).

**Figure 22:**
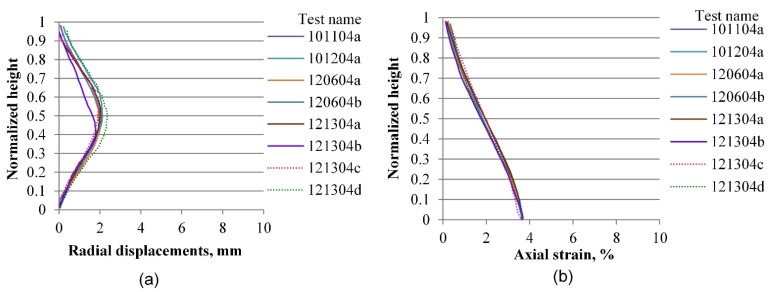
Vertical profiles of averaged displacements at 3.6% of the axial strain of sand specimens prepared by the dry pluviation method: radial displacements field (**a**) and vertical displacements field (**b**).

**Figure 23:**
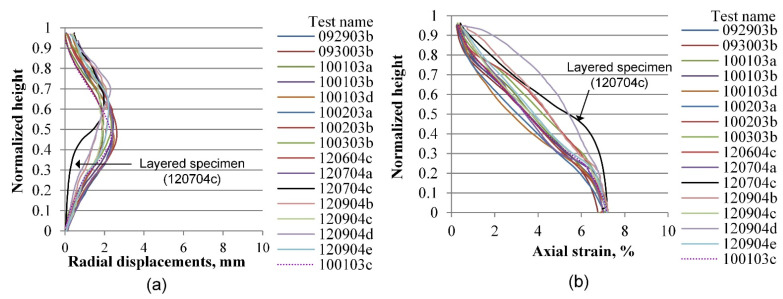
Vertical profiles of averaged displacements at 7.0% of the axial strain of sand specimens prepared by the vibratory compaction method: radial displacements (**a**) and vertical displacements (**b**).

**Figure 24:**
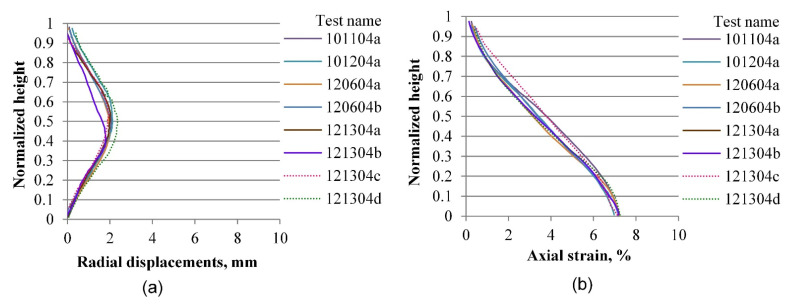
Vertical profiles of averaged displacements at 7.0% of the axial strain of sand specimens prepared by the dry pluviation method: radial displacements (**a**) and vertical displacements (**b**).

**Figure 25:**
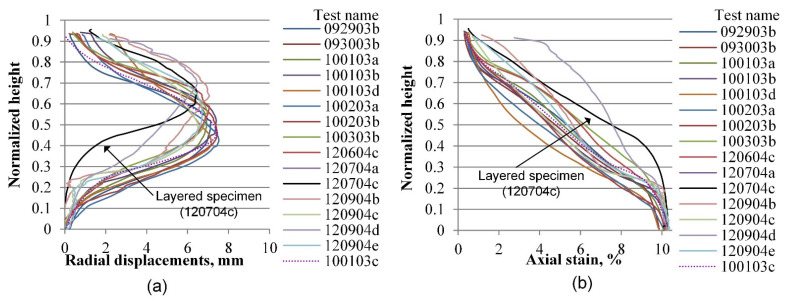
Vertical profiles of averaged displacements at 10.0% of the axial strain of sand specimens prepared by the vibratory compaction method: radial displacements (**a**) and vertical displacements (**b**).

**Figure 26:**
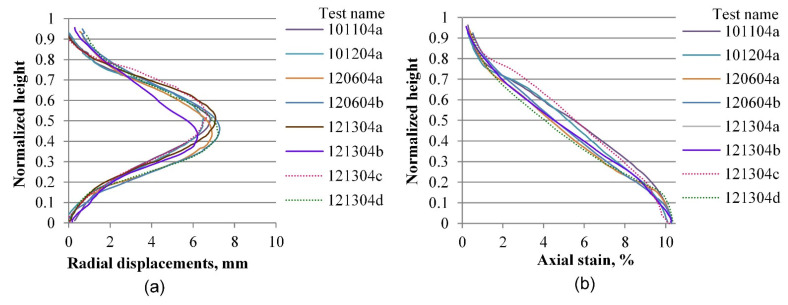
Vertical profiles of averaged displacements at 10.0% of the axial strain of sand specimens prepared by the dry pluviation method: radial displacements (**a**) and vertical displacements (**b**).

The same process as the one described in the previous section is performed for radial and vertical displacements for all tests included in the experimental database, considering four axial strain levels of 0.2, 3.6, 7.0, and 10.0%, which correspond to the elastic range, peak stress, softening zone, and critical state, respectively. Figure 19, Figure 20, Figure 21, Figure 22, Figure 23, Figure 24, Figure 25 and Figure 26 group the results according to the two different sample preparation methods considered for the development of the experimental design (vibratory compaction and dry pluviation), showing the effect of material heterogeneity, as observed thanks to the use of 3D-DIC.

In the elastic range, defined by deformation levels ranging between zero and 0.2% of axial strain as depicted in Figure 19 and Figure 20, there is no indication yet of any localization effects, in either the radial or the vertical displacement profiles. Notice that a preceding set of papers by Medina-Cetina and Rechenmacher focused on the elastic response, including digital image analysis, 2-D and 3-D finite element modeling, and the optimization and probabilistic calibration of these models respectively [17,18,24], thought to serve as a reference to detect at early deformation stages patterns of the most likely local failure mechanisms, now observed at advanced stages of deformation, as discussed below and in the follow-up papers.

When tests progress to the peak deviatoric stress, however, for specimens prepared by the vibratory compaction method, which by design produces heterogeneous specimens (Figure 21), bulging starts to appear around the middle sample height for most specimens, except for the layered specimen (test 120704c), which only expands radially in its loose segment (upper part). This effect is also observed on this test’s vertical displacement profile, where the lower half of the layered specimen behaves like a rigid block compared to the upper segment, suggesting that the overall strength of the specimen depends on the weaker part of its structure. In the other set of specimens prepared by the dry pluviation method, which by design produces homogeneous specimens, both the radial and vertical displacements are almost uniform across all tests (Figure 22), except for the loose specimen (test 121304b), which shows its maximum bulging point shifted to its lower part rather than the middle part of the specimen.

Figure 23 and Figure 24 present the radial and vertical displacement fields when tests progressed into the softening range, where shear bands were developed for most of the specimens. The complexity and variability of the material response become evident at this stage of deformation. Figure 23, for instance, shows the two-layered specimen with a clear and unique radial expansion mode that indicates deformation mainly on its loose upper part, whereas the lower part shows less displacement compared with other specimens. The corresponding vertical profile of the vertical displacements is presented in Figure 23b, which shows a more random behavior due to the specimen heterogeneity. Specimens prepared by the dry pluviation method show a more homogeneous pattern in both radial and vertical profiles (Figure 24). Based on this set of results, we can determine that the dry pluviation method constitutes a specimen with a relatively uniform density distribution compared to a specimen constituted by the vibratory compaction method, where specimens are compacted into three layers, inducing material heterogeneity. This interpretation can also account for the material responses presented in Figure 24b, in which no rigid moving block is seen around the bottom of the specimen because local stiffness is relatively uniform along the height of the specimen.

Figure 25 and Figure 26 show the vertical profiles of the averaged radial and vertical displacement fields at 10.0% of axial strain, which corresponds to the range of critical state. A similar deformation pattern is seen between softening and critical states for all displacement fields; however, variability is higher compared to previous deformation stages. There is no significant change in any of the deformation patterns when comparing tests subjected to different confinement stress, which is consistent with a test dependent on displacement rate and the range of confinement pressures utilized.

### 5.3. Volumetric Strain

**Figure 27:**
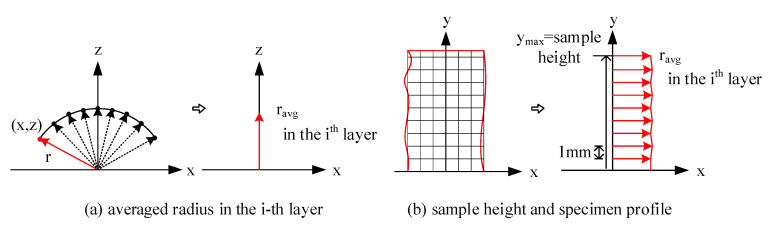
Schematic illustration of averaging radius at each specific height of the specimen.

Because the plexiglass was removed to enable images to be taken during the compression test, the triaxial tubing line used to measure volumetric strain was used to apply the vacuum pressure. Therefore, there is no direct measure of volumetric change. Since boundary measurements were taken during the compression test, however, such measurements can be used to compute the volumetric strain. A method was devised to compute the volumetric strain by considering each specimen’s volume as a sum of a series of “stacked disks.” The height of each disk was determined as a uniform value of 1 mm, and the diameter of each disk was derived from the specimen’s 3-D profile, assumed to be the average radius measured on the area of interest for each “layer,” as shown in Figure 27. This approach has been previously used with a circular disk model to estimate the volume from a digitized image [31]. Results from this technique showed good agreement with conventional volumetric strain measurements obtained in wet conditions.

To compute the volumetric strain of a specimen, a trapezoidal integration function (Equation (1)) was used to estimate the specimen volume at each shearing stage. As stated above, the height of each disk is 1 mm, and the radius of each layer is estimated through the averaged radius captured by 3D-DIC at the corresponding height of the specimen (Figure 27a). Once the volume of each disk is calculated, the ensemble of all volumetric disks represents the bulk volume of the specimen at the current experimental stage.

(1) $$V=\int_{0}^{H}\int_{0}^{2\pi }\int_{0}^{R}r\;\mathrm{dr}\;d\theta \;\mathrm{dz}=\int_{0}^{H}\int_{0}^{2\pi }\frac{1}{2}R^{2}\;d\theta \;\mathrm{dz}=\frac{1}{2}R^{2}2\pi \int_{0}^{H}\mathrm{dz}{=\pi R}^{2}\int_{0}^{H}\mathrm{dz}$$

**Figure 28:**
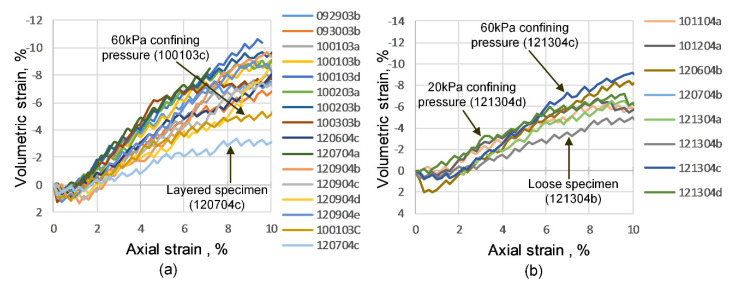
Axial strain versus volumetric strain curves: specimens prepared by vibratory compaction method (**a**) and specimens prepared by the dry pluviation method (**b**).

Figure 28 presents the axial strain versus volumetric strain computed using the disks’ profile as derived from 3D-DIC analysis. This figure shows an overall trend of the first compression and then dilation occurring for most specimens with relatively high density. The maximum compression of data ensemble occurred around 1.0% of axial strain, and most tests reach the critical state close to 9.0% of axial shearing. The two-layered and the loose specimens showed less dilation compared to dense specimens because of their loose composition. The volumetric behavior of layered and loose specimens exhibited high similarity prior to 5.0% of axial strain, implying that the volumetric strain is most important for the specimen’s loose part, as previously noted.

## 6. Conclusions

This paper introduces a comprehensive experimental database, including results of 3-D Digital Image Correlation analyses (3D-DIC) of triaxial compression tests, performed on dry vacuum-consolidated sand specimens. The use of 3D-DIC technology allowed to compute the specimen’s global and local boundary displacement fields, from start to end of the compression phase, in a segment of the specimen’s surface. In addition, standard force-displacement readings were collected from the triaxial standard device. To fully extract the 3-D displacement fields, post-processing analysis consisting of geometric transformation and the integration of sequences of data was performed to use the undeformed configuration at zero axial strain as the reference coordinate system and to accumulate displacements from the step-wise analyses to populate the full displacement field. Results showed different patterns of local deformations, including effects that could be identified visually as bulging and shearing. The displacements’ fields confirmed that sand specimens prepared by the vibratory compaction method had more variability because of their three-layer heterogeneous composition, which can be associated with variations in relative density within the specimen; in contrast, sand specimens prepared by dry pluviation have a more homogeneous structure, which presented much less displacement variability. The two-layer specimen (lower dense, upper loose) also showed localization effects in its upper segment, which is consistent with reduced strength or being the weaker part of the specimen.

Repeatability of a number of tests sheared under the same experimental conditions allowed to identify the effect of heterogeneity on the randomness of the specimen displacement response, which is expected to have an influence on the specimen’s estimation of its mechanical constitutive parameters. A unique set of observations regarding localization phenomena was possible to achieve by the use of a first representation of the 3D-DIC data as vertical profiles of averaged radial and vertical displacement fields, which is suitable for calibrating simple and computationally efficient orthotropic numerical models. The integration of a database, which is being made available to the research community, will allow each test’s global and local deformations to be combined to calibrate and develop forward models, to test hypotheses on the impact of the heterogeneous composition of the specimens, and to account for the displacement fields’ uncertainty propagation. This work will be the basis for statistical characterization of spatio-temporal boundary localization effects to produce stochastic models that generate virtual random simulations of a test’s displacement fields, and that can also be used as a basis for implementing machine-learning algorithms. This will lead to significant opportunities to explore further localization effects tied to global and local deformations at different deformation stages by virtual testing.

## Figures and Tables

**Table 1:** Experimental characteristics of multiple-layered sand specimens prepared by vibratory compaction (heterogeneous specimens).

Test No.	Test Name	Height (mm)	Diameter (mm)	Initial Density (kg/m^3^)	Relative Density (%)	Confinement (kPa)	Notes
1	092903b	155.50	71.33	1710.95	91.09	40	-
2	093003b	156.67	71.41	1696.00	85.96	40	-
3	100103a	157.67	71.29	1702.22	88.10	40	-
4	100103b	155.83	71.24	1717.13	93.18	40	-
5	100103c	157.67	71.54	1703.87	88.67	60	-
6	100103d	154.33	70.86	1702.41	88.17	40	-
7	100203a	157.50	71.45	1715.32	92.57	40	-
8	100203b	155.00	71.48	1711.91	91.41	40	-
9	100303b	158.17	71.29	1718.70	93.71	40	-
14	120604c	158.83	70.72	1717.48	93.30	40	Light reflection
15	120604d	158.83	70.84	1716.99	93.13	40	Light reflection
16	120704a	158.83	71.37	1708.07	90.11	40	Light reflection
17	120704b	159.00	71.30	1686.96	82.82	40	Light reflection
18	120704c	157.67	Average: 70.88	Average: 1648.06	Average: 68.90	40	Layered specimen
		79.50	71.27	1734.17	98.87	40	Lower: dense sand
		78.17	70.68	1549.61	30.54	40	Upper: loose sand
19	120904a	158.67	71.15	1707.72	89.99	40	Light reflection
20	120904b	160.00	70.98	1720.40	94.28	40	-
21	120904c	159.67	71.11	1713.13	91.83	40	-
22	120904d	159.00	71.13	1707.89	90.04	40	-
23	120904e	160.00	70.99	1718.70	93.71	40	-

**Table 2:** Experimental characteristics of single-layer sand specimens prepared by dry pluviation (homogeneous specimens).

Test No.	Test Name	Height (mm)	Diameter (mm)	Initial Density (kg/m^3^)	Relative Density (%)	Confinement (kPa)	Notes
10	101104a	159.33	70.87	1724.89	95.79	40	Light reflection
11	101204a	160.00	71.46	1708.03	90.09	40	-
12	120604a	159.33	71.31	1721.06	94.50	40	Light reflection
13	120604b	159.33	70.94	1715.13	92.50	40	Light reflection
24	121304a	160.00	71.30	1721.73	94.73	40	-
25	121304b	158.17	70.86	1588.84	46.39	40	Loose specimen
26	121304c	160.00	70.48	1718.72	93.72	60	-
27	121304d	159.50	71.38	1736.71	99.71	20	-

## Data Availability

The experimental data supporting the findings of this paper are provided along with the article and made available at the Texas Data Repository (https://dataverse.tdl.org/dataverse/tamu (accessed on 1 December 2021)). Additional queries may be directed to the corresponding author.

## Supplementary Materials

All data used in this paper, including readings from the triaxial device (global response) and boundary displacement fields (local response) as captured by the 3D-DIC, are available at https://dataverse.tdl.org/dataverse/SGL-MDPI-Topic-StochasticGeomechancis-ForwardModeling (last accessed on 1 December 2021).
